# Effectiveness and mechanisms of the arts therapies in forensic care. A systematic review, narrative synthesis, and meta analysis

**DOI:** 10.3389/fpsyt.2023.1128252

**Published:** 2023-05-19

**Authors:** Annemarie Abbing, Suzanne Haeyen, Sashank Nyapati, Peter Verboon, Susan van Hooren

**Affiliations:** ^1^Department of Clinical Psychology, Faculty of Psychology, Open University, Heerlen, Netherlands; ^2^Department of Arts Therapies, Professorship Anthroposophic Healthcare, University of Applied Sciences, Leiden, Netherlands; ^3^KenVaK, Research Centre for the Arts Therapies, Heerlen, Netherlands; ^4^Special Research Group Arts and Psychomotor Therapies/Master of Arts Therapies, Hogeschool van Arnhem en Nijmegen University of Applied Sciences, Nijmegen, Netherlands; ^5^Scelta, Expert Centre for Personality Disorders Apeldoorn, GGNet, Centre for Mental Health, Apeldoorn, Netherlands; ^6^Department of Methodology and Statistics, Faculty of Psychology, Open University, Heerlen, Netherlands

**Keywords:** arts therapies, art therapy, music therapy, drama therapy, dance therapy, forensic, meta-analysis, systematic review

## Abstract

**Introduction:**

Mental health care provided to offenders with psychiatric problems in forensic settings mainly consists of verbal oriented treatments. In addition, experience-based therapies are used such as (creative) arts therapies: (visual) art therapy, music therapy, drama therapy and dance (movement) therapy. There are indications for effectiveness of arts therapies, but a systematic overview of effect studies of all arts therapies in forensic care is lacking.

**Methods:**

First, we performed a systematic review. Second, Thematic Analysis was used to synthesize the qualitative narrative results and define the hypothesized mechanisms of change. Third, we performed a meta-analysis to investigate the effects of arts therapies in reducing psychosocial problems of offenders. Twenty-three studies were included in the review. Quality and risk of bias was assessed using EPHPP (Effective Public Health Practice Project).

**Results:**

The included studies were heterogeneous in type of outcome measures and intervention characteristics. Synthesis of mechanisms of change involved in the methodical use of the arts in arts therapies resulted in a description of regulatory processes which are stimulated in arts therapies: perceptive awareness (interoceptive and exteroceptive), the regulation of emotions, stress, impulses, cognitions, social regulation, and self-expression. These processes play a role in developing prevention, coping and self-management skills. Eighteen studies were included in the meta-analyses (11 RCTs/CCTs; 7 pre-post studies). The meta-analyses indicated significant effects on both risk factors (psychiatric symptoms and addiction) and protective factors for criminal behavior (social functioning and psychological functioning). Effects on criminal and/or antisocial behavior were not significant, but this outcome measure was scarcely used among the studies.

**Discussion:**

The analyses in this study should be considered explorative. More research is needed to gain more solid conclusions about effectiveness and mechanisms of change of arts therapies in forensic institutions. However, the results of this first systematic review, synthesis of mechanisms and meta-analysis in this field are promising and show effects of arts therapies on risk and protective factors in individuals in forensic institutions.

**Systematic review registration:**

https://www.crd.york.ac.uk/prospero/display_record.php?ID=CRD42020217884, identifier: CRD42020217884.

## 1. Introduction

Mental illness plays a role in criminal behavior; many detainees in forensic institutions struggle with a combination of personality problems, aggressive and disruptive behavior, psychosis, mild intellectual disabilities and/or a traumatic history and these problems increase the risk of recidivism (1). Detained individuals with mental health problems often have more than one disorder which for some individuals may impact their behavior and increase their risk of re-offending. Many of these individuals have committed violent crimes and pose an increased risk of danger to society when left untreated. Treatment is provided in several correctional settings. For purposes of this review, correctional settings include forensic institutions and correctional settings where forensic mental health care is provided for detained individuals. Forensic care focuses on understanding and treating mental disorders in offenders and is provided in settings where the individual is involuntary detained. The aim of forensic care is to treat and rehabilitate the patient and to assess risk of recidivism and protect society (2–4). Long-term treatment is often needed, consisting of forensic mental health care and addiction care.

Mental health care in forensic institutions and correctional settings mainly consists of verbal treatments. For various reasons, this specific population may also need treatment interventions that make use of behavioral and experience-oriented techniques (5–8). One of the reasons is that it can be difficult for offenders with mental health problems to gain insight into own behavioral patterns and possible underlying causes when it comes to complex interactions. Other ways than verbal oriented interventions should then be used to help people initiate change, especially when it comes to long-established response patterns and when the range of coping skills needs to be broadened. Then, experiential approaches, including arts therapies, can be considered, which are characterized by learning through action-based experiences and focusing on the “here and now”, guided by a therapist (9). (Creative) arts therapies can offer offenders an experiential insight into their own behavior and how to change it. Arts therapies may promote motivation for treatment and may therefore enhance protective factors against future recidivism (10).

The arts therapies deployed in forensic care are: visual arts therapy (from this point we refer to this modality as “art therapy”), music therapy, drama therapy and dance (movement) therapy. These forms of therapy consist of the psychotherapeutic use of the different art modalities (11–15): visual art, music, play, roleplay, performance, improvisation, dance and movement. Interventions provide kinaesthetic, sensory, perceptual, and symbolic opportunities to invite alternative modes of receptive and expressive communication.

Arts therapies have been used in forensic institutions since the 1950s. The importance of these therapies in forensic and correctional settings is recognized by care providers, offenders with psychiatric problems and policymakers (16–18). With the use of arts therapies interventions, behavioral patterns, limitations and possibilities of patients can become visible and therapy can focus on changes in thinking, feeling and acting. The experience- and action-oriented nature of these interventions makes the arts therapies suitable for descriptive observations that add to the general diagnostics in the forensic setting [e.g., (19, 20)] and for working on treatment goals such as tension regulation, improving impulse control, aggression regulation, developing empathy and improving social interaction. Other options are exploring one's own identity and improving the often negative self-image (21). Because of the non-verbal character, the patient does not have to speak about feelings and thoughts when they are still too threatening. Even with a low level of intellectual functioning and problems in verbal communication, the arts therapies can offer an experiential entrance to explore thoughts and feelings (22). Furthermore, arts therapies can offer the opportunity to mentally escape from the restrictive environment for a while and it can provide space for the (controlled) expression of emotions, whereby the intensity of the emotions (such as aggression) can be calmed down (21). The arts therapies can also have a training-oriented character, whereby new behavior can be practiced, which is a prerequisite for breaking the crime chain (23, 24).

Although arts therapies are widely used in forensic care, there is still little insight into the effectiveness and associated mechanisms of change of the arts therapies in forensic psychiatry because of the scarce research on this topic. Only three systematic reviews on this topic have been performed that are related to this topic, but two of these reviews were aimed at psychological therapies and not exclusively on arts therapies (25, 26) and one concerned solely music therapy (27). Despite several methodological shortcomings of the included studies in these reviews, the conclusions were that psychological (arts) interventions can have positive effects in prisons or forensic care. Main effects were improved mental health and better coping with emotions and feelings. It was indicated that the outcomes could be linked to better anger management and improved empathy, important outcomes for the forensic setting. Also, increasing self-confidence and social functioning were mentioned as effects of music therapy. From these reviews can be concluded that there are indications for effectiveness of arts therapies, but a systematic overview of effect studies of all arts therapies in forensic care is lacking.

To gain insight in the effectiveness of arts therapies in forensic institutions we focused on the following research questions: And what are the hypothesized mechanisms of change of the arts therapies in forensic care? And are arts therapies effective in reducing psychosocial problems of detained adults in forensic institutions? Are these effective in terms of protective or risk factors?

## 2. Methods

### 2.1. Protocol and registration

The review protocol for this systematic review and meta-analysis was registered at Prospero, number CRD42020217884 (28). The recommendations of the Cochrane Collaboration were followed, concerning study identification, selection, data extraction, quality appraisal and data analysis (29). The PRISMA Guidelines were used for reporting (30) and PRISMA Checklist was used (Supplementary material 8).

### 2.2. Search strategy

Multiple systematic searches were performed with the help of a medical information specialist (TP) from inception until June 2020 in the following databases: PubMed, CINAHL plus with full text (EBSCO), APA PsycInfo (EBSCO), Web of Science Core Collection and EMBASE (OVID). The following terms were used (including synonyms and closely related words) as index terms or free-text words to represent the following concepts: arts therapies (art therapy, music therapy, drama therapy, and dance movement therapy) AND forensic OR correctional setting AND effect study. No additional filters were used. The full searches are available in the appendices (Supplementary material 1). Per database all results were exported into a single file. Next, all files were merged and de-duplicated using EndNote. The bibliographies of included studies were hand searched for further relevant studies. Bibliographies of reviews on arts therapies in forensic care were hand searched as well to identify other relevant studies.

### 2.3. Study selection

Our research design was restricted to peer reviewed published effect studies with at least pre and post-test. Included were Randomized Controlled Trials (RCTs), Controlled Clinical Trials (CCTs), case control studies, cohort studies and multiple case studies, published in English, Dutch or German language, from 1980 until June 2020. The types of participants and setting were restricted to detained individuals with mental health problems in a correctional setting or a forensic institution, with or without mental health services, aged 18–65 years, from any gender or ethnicity. The studies investigated arts therapies: art therapy, music therapy, drama therapy and dance movement therapy, provided by an arts therapist, to individuals or groups with no limitations on duration or number of sessions. Interventions without a trained arts therapist were excluded. A trained arts therapist has received a bachelor of master educational program in the field of one of the arts therapies. We included studies with the following types of comparison: (1) Arts therapies vs. no treatment/waiting list, (2) Arts therapies vs. inactive control, (3) Arts therapies vs. active control (e.g., CBT), (4) No control. The type of outcome measures were restricted to quantitative measures of psychosocial outcomes.

### 2.4. Selection process

Duplicate studies were removed and the references were imputed in Rayyan (www.Rayyan.QCRI.org). Three researchers independently performed study selection and recorded their decisions in Rayyan QCRI in two separate rounds: (1) screening the titles and abstracts of the included titles and (2) subsequently reading and evaluating the full texts of the included articles. The researchers were blinded for decisions of other reviewers. Conflicts between scores of reviewers were resolved via discussion or by consulting a fourth reviewer.

### 2.5. Data collection process

The data collection process was performed by four researchers (AA, SH, RK, VB) who separately extracted the data from included publications using a standard extraction sheet (Supplementary material 2). Coding was replicated by one of the researchers (SN).

### 2.6. Quality assessment

The Quality Assessment tool for Quantitative studies (31) was used for assessing study quality and risk of bias. To reach a conclusion, Quality Assessment Tool uses a number of factors along with opinions of select experts who meet pre-determined criteria. Once the assessment is fulfilled, each examined practice receives a mark ranging between “strong,” “moderate,” and “weak” in eight categories: Study design, Analysis, Withdrawals and dropouts, Data collection practices, Selection bias, Invention integrity, Blinding as part of a controlled trial, and Confounders.

### 2.7. Coding

Outcome measures of the various studies were clustered according to a predetermined set of clusters, based on the Risk-Needs-Responsivity model (32), the Good Lives Model (33), and the HKT- R (34). Clustering was made based on risk factors and protective factors.

Risk factors: (1) Criminal and antisocial behavior (aggression, anger, hostility, impulsivity, incidents, and recidivism); (2) Addiction (substance abuse, behavior, and thinking styles related to addiction); (3) Psychiatric symptoms (e.g., psychosis, PTSD, anxiety, depression, and personality disorders).

Protective factors: (4) Social and relational functioning (interaction and social behavior, social regulation, empathy, attachment, and trust); (5) Psychological functioning (processes and skills related to internal regulation, such as coping, insight, motivation, emotions, mood, relaxation, body awareness, attention, control, self-confidence, self-image, quality of life, and general wellbeing).

### 2.8. Analyses

#### 2.8.1. Narrative synthesis

A narrative synthesis of the arts therapies interventions and proposed mechanisms of change was made. In this synthesis, a mechanism of change was defined as the (components of) the process by which an effect seems to be produced (9). Parts of the text were selected in which the authors interpret the results of their study by means of supposed mechanisms. These texts were summarized and clustered per modality. Based on Thematic Analysis (35), the hypothesized mechanisms of change were clustered for arts therapies in total.

#### 2.8.2. Meta-analysis

##### 2.8.2.1. Data RCT design

First all records with design RCT or CCT were selected.

The standardized mean difference (36) effect size (“SMD”) and variance of the effect size were computed using the escalc function from the metafor package (37) and these variables were added to the data.

##### 2.8.2.2. Data pre-post design

First all records with pre-post design were selected. The correlation (*r*) between the two measurements was never reported. The value of *r* = 0.5 was therefore used as a reasonable approximation. Next, based on the means and SD's of the experimental group at T1 and T2 and the correlation between them, an effect size (“SMCC”) and variance of the effect size were computed using the escalc function from the metafor package (37). The “SMCC” is the standardized mean change using change score standardization (38).

In both datasets, all effects were coded such that a positive effect size implies an effect in accordance with the expected effect of the intervention.

##### 2.8.2.3. Meta-analysis on the two datasets

Meta-analyses with random effects using metafor package were run on these two datasets. First, on the total sample and subsequently with moderators intervention type, and setting [forensic (psychiatric) care and forensic/correctional] to determine whether or not it makes a difference which form of arts therapies is deployed and in which setting. IQ was included in data-extraction. Additional meta-analyses with random effects were run for risk factors and protective factors separately, followed by meta-analyses of each category of risk and protective factors separately. For each analysis, heterogeneity was explored by calculating the *I*^2^ statistics. Thresholds for the interpretation of *I*^2^ can be misleading, since the importance of inconsistency depends on several factors. A rough guide to interpretation is as follows: 0–40%: might not be important; 30–60%: may represent moderate heterogeneity; 50–90%: may represent substantial heterogeneity; 75–100%: considerable heterogeneity (29).

Analysis scripts are included as Supplementary materials 3, 4.

## 3. Results

### 3.1. Study selection

**Figure 1:**
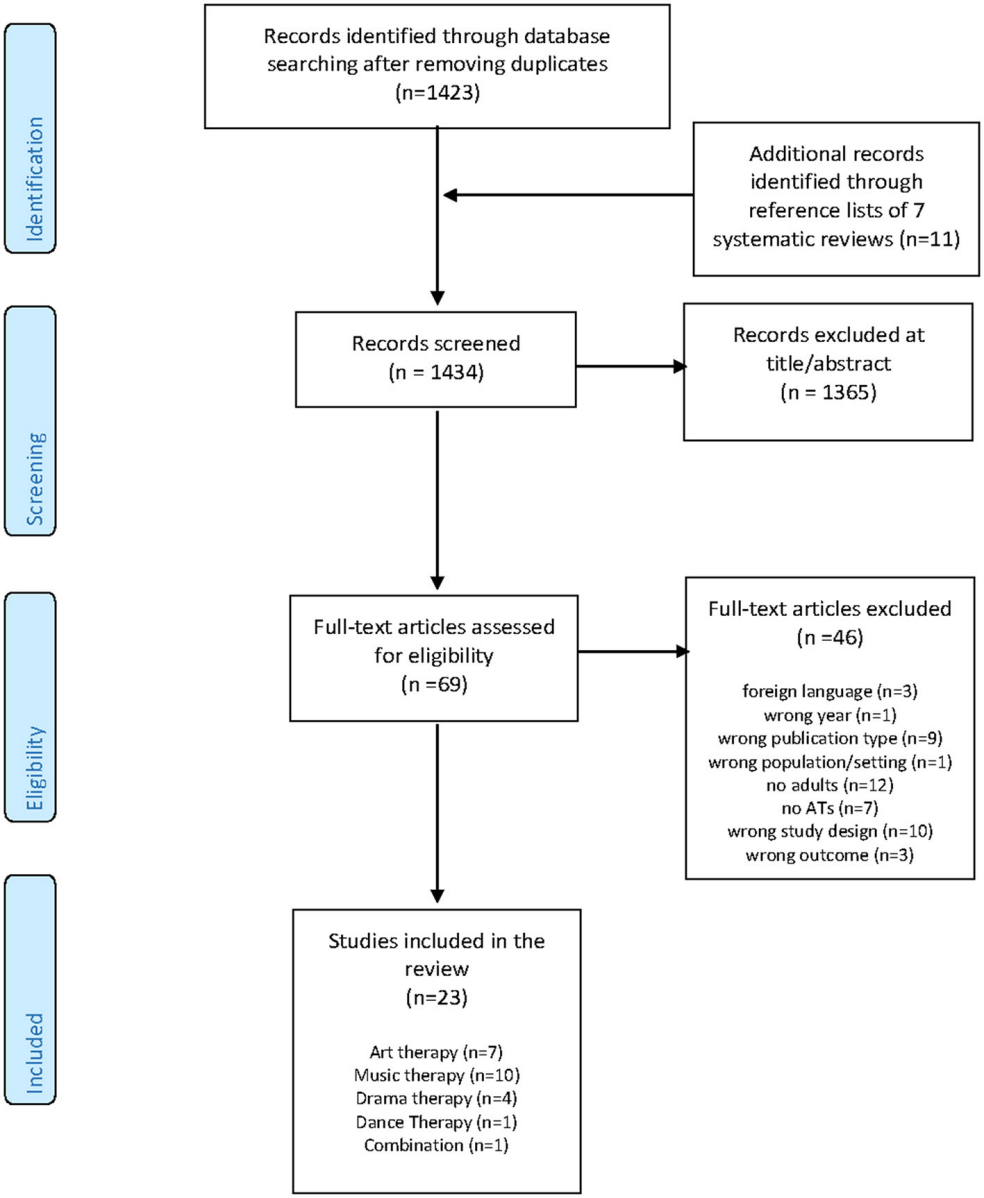
PRISMA flow diagram.

The search resulted in 1,423 unique citations (see Figure 1). The search yielded seven systematic reviews. Screening of the titles of the reference lists of these reviews resulted in 11 extra citations that were eligible for further screening. Based on title and abstract, 1,365 citations were excluded based on the inclusion and exclusion criteria. The 69 remaining full-text original research articles were screened for eligibility. Forty-six articles were excluded. See Supplementary material 5 for references and reasons to exclude studies and outcomes. Twenty-three studies were included in the review.

### 3.2. Study characteristics

The 23 included effect studies on arts therapies in forensic care included 1,118 offenders in total with sample sizes ranging from 7 to 200. An overview is presented in Table 1, outcomes from studies are reported in Supplementary material 2.

**Table 1:** Characteristics and quality of studies investigating arts therapies in forensic care.

	**References**	**Design**	**Quality**	**Total *N***	**Population**	**Setting**	**Intervention**	**Control**
1	Gussak (44)	Pre-post pilot	Medium	39	Male inmates with Axis I diagnosis	FC^*^	Art therapy—*directive*	None
2	Gussak (39)	RCT	Medium	39	Male inmates with Axis I diagnosis	FC	Art therapy—*directive*	Yes—no intervention
3	Gussak (45)	Pre-post/follow-up with control	Weak	39	Male inmates with Axis I diagnosis	FC	Art therapy—*directive*	
4	Qiu et al. (40)	RCT	Weak	105	Male/female inmates with schizophrenia	FC	Art therapy—*non-directive*	Yes—CAU
5; 6	Gussak (42, 43)^**^	CCT	Weak	159	Male/female inmates	F^*^	Art therapy—*directive*	Yes—Waitlist
7	Yu et al. (41)	RCT	Medium	72	Male inmates with pre-release anxiety	F	Art therapy—*directive*	Yes—Other Intervention
8	Hakvoort et al. (50)	Pre-post	Weak	12	Male forensic psychiatric patients within their first one and a half years of forensic hospitalization	FC	Music therapy-*active*	Yes—CAU
9	Jeon et al. (51)	CCT	Strong	38	Male prisoners with schizophrenia	FC	Music therapy-*active*	Yes—CAU
10	Kellett et al. (52)	CCT	Weak	20	Male forensic patients with schizophrenia or other serious mental disorder	FC	Music therapy—*active and receptive*	Yes—CAU
11	Macfarlane et al. (53)	Pre-post single case	Weak	16	Male inmates with PTSD	FC	Music therapy-*active*	None
12	Thaut (54)	Pre-post	Weak	50	Male inmates with psychiatric diagnosis	FC	Music therapy—*active and receptive*	None
13	Van Alphen et al. (48)	RCT	Strong	35	Psychiatric inmates with severe attention problems	FC	Music therapy—*active and receptive*	Yes—CAU
14	Zeuch and Hillecke (49)	Pre-post	Weak	11	Male delinquents in a social therapeutic setting	FC	Music therapy—*receptive*	None
15	Chen et al. (27)	Pre-post	Strong	200	Male inmates with high anxiety and depression scores	F	Music therapy-*active*	Yes—CAU
16	Gold et al. (55)	RCT	Medium	113	Male inmates	F	Music therapy-*active*	Yes—CAU
17	Gold et al. (56)	Pre-post	Medium	66	Male inmates	F	Music therapy-*active*	Yes—CAU
18	Keulen-de Vos et al. (57)	Observational design	Weak	9	Male inmates with personality disorders	FC	Drama therapy—*psychodrama*	None
19	Morris and Moore (58)	Pre-post	Weak	22	High secure male patients with substance abuse	FC	Drama therapy—*psychodrama*	None
20	Stallone (59)	CCT	Medium	66	Male inmates in a voluntary intensive treatment program	F	Drama therapy—*psychodrama*	Yes—No intervention
21	Testoni et al. (60)	Mixed method	Weak	7	Addicted male inmates	F	Drama therapy—*psychodrama*	None
22	Koch et al. (46)	CCT	Medium	47	Male prison inmates	F	Dance movement therapy *with martial arts*	Waitlist
23	Van den Broek et al. (47)	RCT	Strong	10	Male forensic patients with cluster B personality disorders	FC	Drama therapy/art therapy/PMT	Yes—CAU AND other intervention

* FC, forensic (psychiatric) care setting; F, forensic setting (correctional); CAU, care as usual.

** Studies report same data of one CCT.

#### 3.2.1. Art therapy

The seven included studies were three RCTs (39–41), two studies on one CCT (42, 43), and two pre-post designs (44, 45) with 29–158 participants (414 in total). Four studies concerned male and female prisoners, three studies only male prisoners. Four studies took part in forensic care settings and, three studies included correctional settings, involving inmates without a psychiatric diagnosis but with psychological problems.

#### 3.2.2. Music therapy

Ten studies were included: two RCTs, one CCT and seven pre-post designs without a control group, with 11–200 participants (561 in total). Participants were mostly male inmates with various psychiatric problems. Seven studies concerned a forensic care setting and three studies took place in a forensic, non-psychiatric setting.

#### 3.2.3. Drama therapy

Four studies were included: one CCT and three studies with a single group pre-post design with a range of 7–66 participants (104 in total). Two studies concerned a forensic care setting and two a forensic/correctional setting with solely males. Psychiatric problems were personality disorders (antisocial, bipolar, and narcissistic) and substance abuse/addiction.

#### 3.2.4. Dance movement therapy

One study was included, with a CCT design and 47 male prison inmates (46).

#### 3.2.5. Combination of arts therapies

One RCT pilot study (47) with 10 male offenders with cluster B personality disorders in a forensic care setting.

### 3.3. Methodological quality of the studies

Of the 23 studies, 5 studies had strong methodological quality, seven medium quality and 11 weak quality (Table 1). Key limitations included designs with the lack of a control condition; the use of self-developed measurement instruments and/or poor psychometric quality; small study populations also often heterogeneous in nature; a relatively high dropout rate during the studies. Details of the quality assessment can be found in Supplementary material 6.

### 3.4. Narrative synthesis

#### 3.4.1. Arts therapies interventions characteristics

The number of sessions of all studies varied from 1 to 48 sessions, of 30–90 min per session and with a duration from 1 to 52 weeks. Interventions were offered once or sometimes twice weekly. One intervention was offered 5 days in a row (46). Interventions concerned both group therapy and individual therapy. Art therapy interventions were mostly based on a directive approach with structured art exercises guided by the art therapist. Music therapy mostly involved active music therapy in a group setting, using musical rhythms, singing, song writing, and improvisation. Two studies involved receptive music therapy; listening to recorded music or to pre-played music (48, 49). The drama therapy interventions were mostly based on psychodrama techniques; (realistic) role plays in a group based on situations from past and present. The dance movement intervention was based on Aikido stick fighting.

#### 3.4.2. Hypothesized mechanisms of change

##### 3.4.2.1. Art therapy

Several supposed mechanisms of change were described. First, art therapy is thought to create the possibility to express emotions, distress, impulses, and individual identity [(39–41, 44, 45)]. In this regard, the artistic process could be an acceptable emotional escape [(39, 40, 44, 45)]. The process of creative expression was also seen as an appropriate mean of self-expression, a status builder and a process to earn respect and friendship [(42, 43, 45)]. Second, elements in the art making process (using more space, detail, and compositional integration) may stimulate awareness of surroundings and how clients relate to their surroundings. This may lead to an improved attitude and increased acceptance of each other and the environment, which may result in improved interaction with peers and staff [(40, 42–44)]. Third, the art making process itself had a regulatory and positive effect on mood. Participants enjoyed the art making process and were amazed at their end results, which may have improved their self esteem (39, 41–45). Fourth, the art making process is self-directing and undisturbed, which may allow prisoners to regain their sense of self-control (41). Fifth, the artwork may shed light on their inner world in a concrete way, which could therefore be acknowledged and symbolically embodied in artwork [(40, 41)]. In this way, negative feelings can be transformed into a motivating force, regain courage and confidence (41). In group discussions, the artwork served as a useful mean for reflection [(40, 41)], e.g., on anxious feelings toward life after release (41) or on life events (40). The (group) discussion on artwork stimulated prisoners to discover and formulate insights in cognitions and behavior; the therapist guided this process toward helpful cognitions and behavior. Also, working together artistically in a group created a sense of belonging to the group, stimulated supportive interactions and stimulated social acceptance. At last, the group art activities would help to increase clients' abilities to cooperate and solve problems (44).

##### 3.4.2.2. Music therapy

Several mechanisms of change were described for music therapy. First, music came forward as calming the stress response, which is explained by the incorporation of abdominal breathing techniques, rhythmical entrainment, bilateral movement patterns in music with body percussion, and musical attention control training (53). Furthermore, alternation of tension and relaxation through variously implementing strong and weak drumming and fast and slow tempos may allow exploration of tension, negative emotions and impulsiveness in a positive way (51). Second, music supported to express, identify, and explore emotions in a positive way during music making in which emotional and physiological responses could be elicited and thereby be a mean to identify, explore and eventually influence emotional experiences. This is could lead the way toward insight, improving mood, self-awareness, and (self-) acceptance (48, 49, 54). Third, structured music making or listening can optimize attention processes and this is a proposed mechanism toward inducing symptom reduction. Fourth, music therapy offered practice for using other coping skills and learning impulse regulation skills (50). Also, music can support social processes, by making music in a group which necessitates working together and can strengthen relationships. In individual expression, music could stimulate feelings of autonomy (52, 61).

##### 3.4.2.3. Drama therapy

Dramatherapy offered the opportunity to experiment, to find out more about a client's feelings and thoughts through play. The proposed mechanism of role-play and mirroring in drama therapy lies in self-reflection and thereby increasing awareness of problematic behaviors, prevention of this behavior, promoting ability to empathize and developing solution skills (58), which may increase self-esteem and improve relational attitudes. Roleplay also provided understanding and insight by dramatizing past, present, life situations and roles (59). Drama therapeutic techniques like enactment, playing multiple parts of the self, role reversal, doubling, soliloquy, and future projection (62–64) offered preparation for situations, in prison and when released (59). These drama roles assist behavior change and self-expression.

##### 3.4.2.4. Dance movement therapy

DMT uses a wide range of movement-oriented interventions. In the included study, **t**he mechanism of constant changing roles of the inmate (perpetrator vs. victim) to reactivate situations where violent behavior was executed/encountered. The dance-based Aikido stick fighting techniques combined with role play and body psychotherapeutic interventions sensitized inmates to perceive psychosomatic changes of self and others. The practicing of alternative behaviors helped to develop constructive handling of emotions and aggression, and improved social skills and empathy.

##### 3.4.2.5. Combination of arts therapies

Van den Broek et al. (47) described the experiential character and the potential of the arts therapies to trigger emotional states and spontaneity through the use of artistic media and experiential exercises. Evoking emotional states can help offenders who are emotionally detached to open up for therapeutic processes (47).

More detailed information about intervention characteristics and hypothesized mechanisms of change can be found in Supplementary material 7.

#### 3.4.3. Synthesis of narrative results

The arts therapies, how different they may be in method, theoretical basis and implementation, share overarching hypothesized mechanisms of change. The different arts therapies are described to be working through a number of regulatory processes, shaped through the specific creative means of the arts therapy. These regulatory processes take place on a base of perceptive awareness, which includes both interoceptive and exteroceptive awareness. The regulatory processes include: - stress regulation (calming and relaxation); - impulse regulation (e.g., handling aggression); - emotion regulation (e.g., mood improvement, handling negative emotions, and recognition); - cognitive regulation (mindful attention, stimulating reflection, and helpful cognitions); - social regulation (stimulation of social attunement, working together, acceptance, and empathy) and the process of self-expression. Based on this layer of awareness and regulation, behavior change could be focused on developing prevention skills, social and coping skills, and improved self-management (developing autonomy, identity, empowerment, and wellbeing).

### 3.5. Meta-analysis

For meta-analysis, five studies were excluded. Gussak (45) was excluded because the study used the same data as Gussak (39). The studies by Gussak (43), Van den Broek et al. (47), Gold et al. (56), and Keulen-de Vos et al. (57) were excluded because these studies did not meet the criteria for an effect study with pre-and-post measurements aimed at measuring (possible) symptom reduction. For detailed exclusion reasons, see Supplementary material 5.

From the included studies, some outcomes were excluded for meta-analysis, such as follow-up measures (52), non-validated measurements (39, 52) and combined outcomes of multiple intervention groups (54).

Dataset is available at Mendeley Data, doi: 10.17632/xjpvfjznvc.1 (65).

#### 3.5.1. Results meta analysis RCT/CCT designs

There were 11 studies included in the meta-analysis, which accounted for 95 outcomes/records in this data set with non-missing effect sizes (see Table 2a). The number of effect sizes within each study ran from 0 to 25. Excluded from the analysis were measures that were not validated, observer measures and re-used data.

**Table 2a:** Types per intervention type; RCT/CCT studies.

	**Risk factors**			**Protective factors**	
	**Criminal/antisocial**	**Addiction**	**Psychiatric symptoms**	**Social functioning**	**Psychological functioning**
**Art therapy**					
*4 studies*	3 outcomes	0 outcomes	14 outcomes	5 outcomes	1 outcome
23 outcomes					
**Music therapy**					
*5 studies*	0 outcomes	0 outcomes	14 outcomes	20 outcomes	11 outcomes
45 outcomes					
**Drama therapy**					
*1 study*	0 outcomes	0 outcomes	0 outcomes	2 outcomes	0 outcomes
2 outcomes					
**Dance therapy**					
*1 study*	8 outcomes	0 outcomes	4 outcomes	4 outcomes	8 outcomes
24 outcomes					
**TOTAL**					
*11 studies*	11 outcomes	0 outcomes	32 outcomes	31 outcomes	20 outcomes
94 outcomes					

The first analyses were done on the complete sample. First without moderators and in addition, intervention type and setting (forensic-psychiatric and forensic) were separately included as moderators. On the complete sample, the mixed-effects model (*k* = 95), with a moderate between-study heterogeneity [*Q*_(df = 94)_ = 165.397, *p* < 0.001, *I*^2^ = 46.926%], showed significant effects [*b* = 0.209, 95%CI (0.127–0.290), *SE* = 0.042, *p* < 0.001]. The moderator analyses on type of arts therapies and settings resulted in a mixed-effects model (*k* = 95), with a moderate between-study heterogeneity [*Q*_(df = 91)_ = 138.353, *p* = 0.002, *I*^2^ = 38.62%], showed significant effects for art therapy [*b* = 0.395, 95% CI (0.259, 0.530), *SE* = 0.069, *p* < 0.001] and music therapy [*b* = 0.189, 95% CI (0.077, 0.300), *SE* = 0.057, *p* < 0.001], but not for drama therapy [*b* = 0.777, 95% CI (−0.210, 1.764), *SE* = 0.504, *p* = 0.123] and dance therapy [*b* = −0.026, 95% CI (−0.188, 0.137), *SE* = 0.083, *p* = 0.757]. The mixed-effects model (*k* = 95) for setting had moderate between-study heterogeneity [*Q*_(df = 93)_ = 158.954, *p* < 0.001, *I*^2^ = 44.58%] and showed significant effects for all settings: forensic care settings [*b* = 0.290, 95% CI (0.178, 0.401), *SE* = 0.057, *p* < 0.001] and /correctional settings [*b* = 0.122, 95% CI (0.007, 0.238), *SE* = 0.059, *p* = 0.038].

Next, a random effects meta-analysis (*k* = 94) with moderators was examined. The mixed-effects model showed a significant effect on risk factors [*b* = 0.185, 95% CI (0.072, 0.297), *SE* = 0.057, *p* < 0.01] and on protective factors as well [*b* = 0.235, 95% CI (0.114, 0.357), *SE* = 0.062, *p* < 0.001]. The between-study heterogeneity was moderate [*Q*_(df = 92)_ = 165.062, *p* < 0.001, *I*^2^ = 47.619%]. Subsequently, analyses were done for each category of the risk and protective factors separately. As IQ measures were present in only one of the studies (52), we could not use this as a factor in the meta analysis.

##### 3.5.1.1. Criminal/antisocial

**Figure 2:**
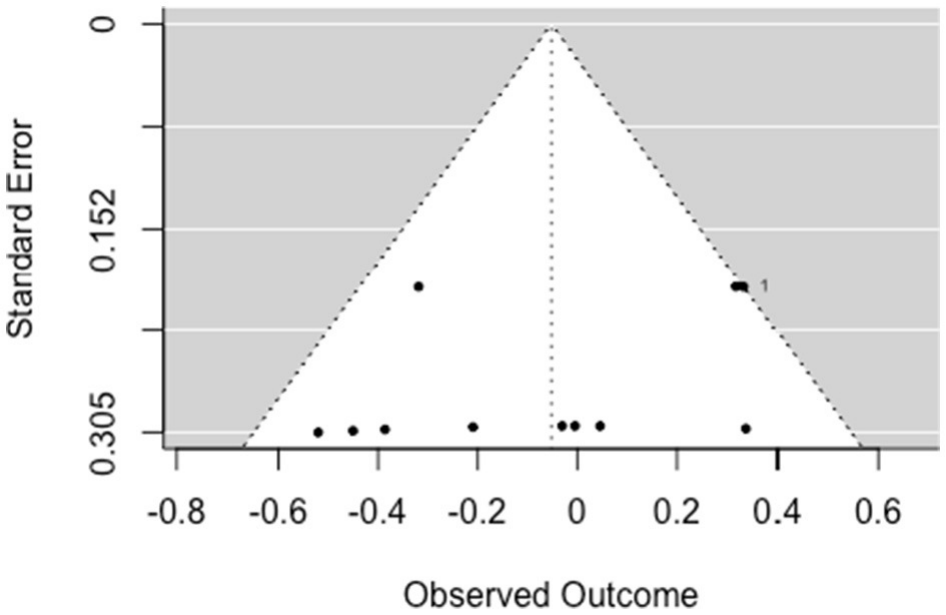
Funnel plot for criminal/antisocial outcomes (RCT/CCT studies). Effect sizes outside the 95% confidence interval around 0 are labeled with their row number in the data.

A multilevel, random-effects meta-analysis (*k* = 11) indicated a non-significant and small negative experimental effect on antisocial behavior [*b* = −0.052, 95% CI (−0.255, 0.151), *SE* = 0.104, *p* = 0.615]. The between-study heterogeneity was moderate [*Q*_(df = 10)_ = 16.509, *p* = 0.086; *I*^2^ = 40.77%]. Based on visual inspection there was no evidence of funnel plot asymmetry (Figure 2). Egger's test gave *z* = −1.3 (*p* = 0.192), which indicated no funnel plot asymmetry.

##### 3.5.1.2. Psychiatric symptoms

**Figure 3:**
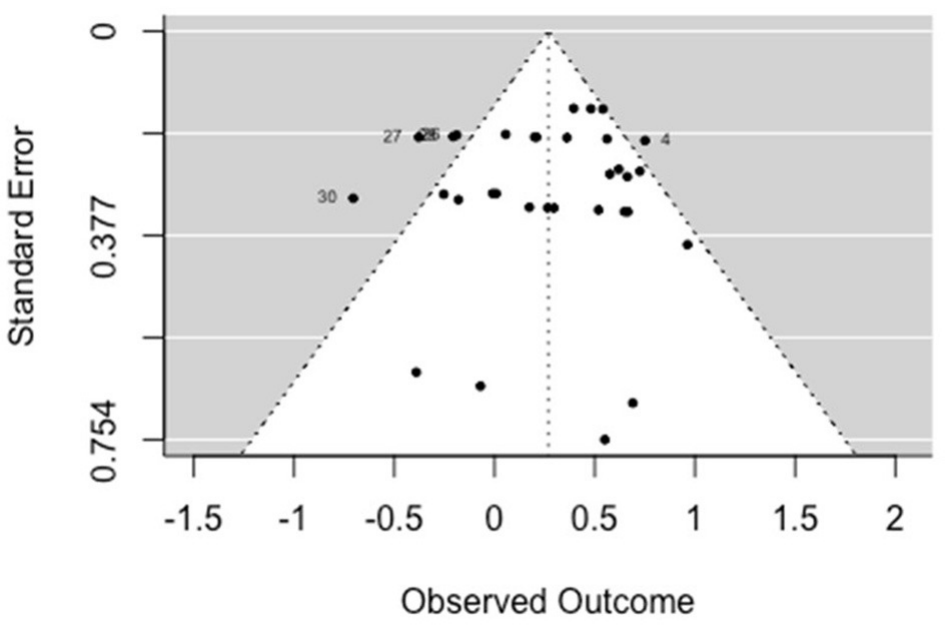
Funnel plot for psychiatric symptoms (RCT/CCT studies). Effect sizes outside the 95% confidence interval around 0 are labeled with their row number in the data set.

The multilevel random-effects meta-analysis (*k* = 32) indicated an overall significant effect on psychiatric symptoms [*b* = 0.268, 95% CI (0.130, 0.406), *SE* = 0.070, *p* < 0.001]. The between-study heterogeneity was moderate [*Q*_(df = 31)_ = 72.013, *p* < 0.001; *I*^2^ = 58.78%]. Based on visual inspection there was no evidence of funnel plot asymmetry (Figure 3). Egger's test gave *z* = −0.11 (*p* = 0.914), which indicated no funnel plot asymmetry.

##### 3.5.1.3. Social-functioning

**Figure 4:**
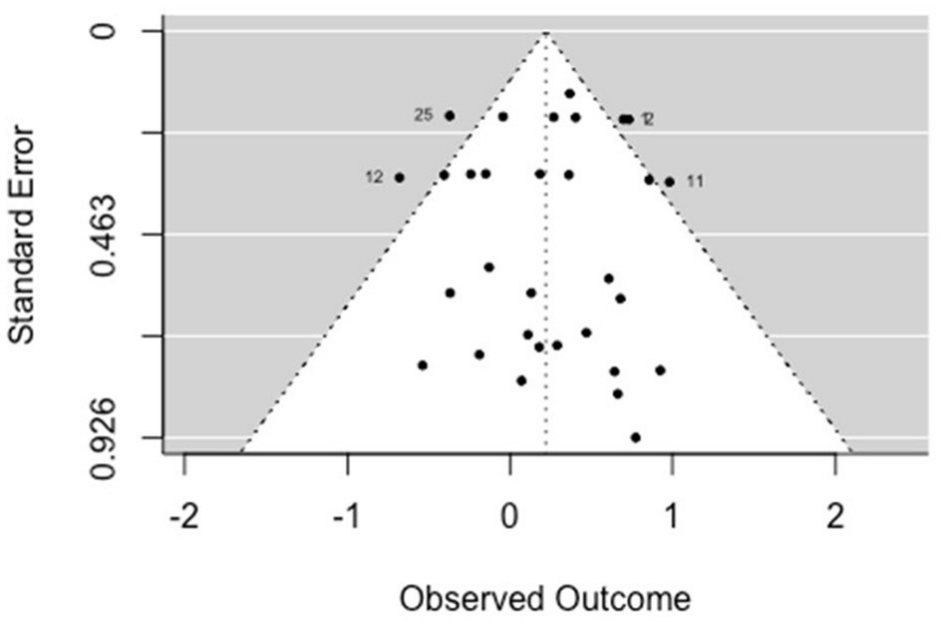
Funnel plot for social functioning (RCT/CCT studies). Effect sizes outside the 95% confidence interval around 0 are labeled with their row number in the data set.

The multilevel random-effects meta-analysis (*k* = 31) indicated an overall significant effect on social functioning [*b* = 0.22, 95% CI (0.036, 0.403), *SE* = 0.094, *p* = 0.019]. The between-study heterogeneity was moderate [*Q*_(df = 30)_ = 53.840, *p* < 0.005; *I*^2^ = 50.23%]. Based on visual inspection there was no evidence of funnel plot asymmetry (Figure 4). Egger's test gave *z* = −0.06 (*p* = 0.956), which indicated no funnel plot asymmetry.

##### 3.5.1.4. Psychological-functioning

**Figure 5:**
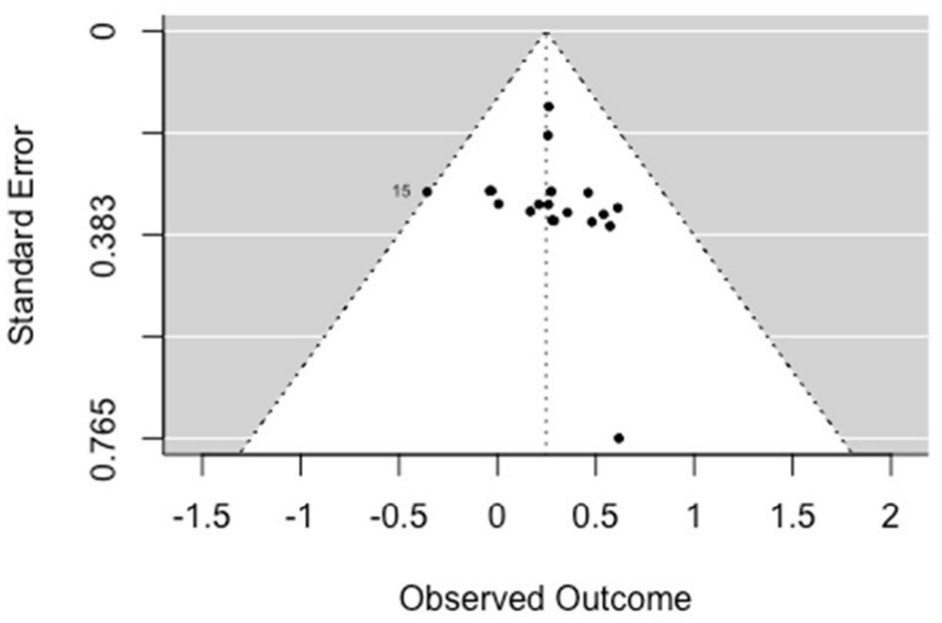
Funnel plot for psychological functioning (RCT/CCT studies). Effect sizes outside the 95% confidence interval around 0 are labeled with their row number in the data set.

The multilevel random-effects meta-analysis (*k* = 20) indicated an overall significant effect on psychological functioning [*b* = 0.247, 95% CI (0.119, 0.374), *SE* = 0.065, *p* < 0.001]. The between-study heterogeneity was very low [*Q*_(df = 19)_ = 10.391, *p* = 0.943; *I*^2^ = 0.00%]. Based on visual inspection there was no evidence of funnel plot asymmetry (Figure 5). Egger's test gave *z* = 0.44 (*p* = 0.659), which indicated no funnel plot asymmetry.

#### 3.5.2. Results meta-analysis of pre-post designs

Seven studies were included in this meta-analysis, with the number of effect sizes within each study running from 0 to 11. After close inspection 7 outcomes were removed, because these concerned non-validated or observer measures. For detailed information about exclusion reasons, see Supplementary material 5.

There were 51 records in this data set with non-missing effect sizes (Table 2b).

**Table 2b:** Types per intervention type; pre-post studies.

	**Risk factors**			**Protective factors**	
	**Criminal/antisocial**	**Addiction**	**Psychiatric symptoms**	**Social functioning**	**Psychological functioning**
**Art therapy**					
*1 study*	0 outcomes	0 outcomes	0 outcomes	6 outcomes	1 outcome
7 outcomes					
**Music therapy**					
*4 studies*	0 outcomes	0 outcomes	1 outcome	0 outcomes	26 outcomes
30 outcomes					
**Drama therapy**					
*2 studies*	0 outcomes	8 outcomes	4 outcomes	0 outcomes	5 outcomes
16 outcomes					
**TOTAL**					
*7 studies*	0 outcomes	8 outcomes	5 outcomes	6 outcomes	32 outcomes
51 outcomes					

The first analyses were done on the complete sample. First without moderators and in addition, intervention type and the setting (forensic-psychiatric, forensic, and other) were separately included as moderators. On the complete sample, the mixed-effects model (*k* = 51), with a considerable between-study heterogeneity [*Q*_(df = 50)_ = 261.981, *p* < 0.001, *I*^2^ = 84.12%], showed significant effects [*b* = 1.019, 95%CI (0.817, 1.270), *SE* = 0.103, *p* < 0.001].

The moderator analyses on type of arts therapies and settings resulted in a mixed-effects model (*k* = 49), with a substantial between-study heterogeneity [*Q*_(df = 46)_ = 175.458, *p* < 0.001, *I*^2^ = 74.95%], showed significant moderation effects [*Q*_(df = 3)_ = 180.000, *p* < 0.001] with significant outcomes for all included arts therapies: art therapy [*b* = 2.150, 95% CI (1.723, 2.577), *SE* = 0.218, *p* < 0.001], music therapy [*b* = 0.906, 95% CI (0.677, 1.135), *SE* = 0.117, *p* < 0.001] and drama therapy [*b* = 0.696, 95% CI (0.412, 0.979), *SE* = 0.145, *p* < 0.001].

The mixed-effects model (*k* = 49) for setting had considerable between-study heterogeneity [*Q*_(df = 47)_ = 261.672, *p* < 0.001, *I*^2^ = 85.40%], showed significant moderation effects [*Q*_(df = 2)_ = 89.601, *p* < 0.001] with significant effects for both settings: forensic care settings [*b* = 1.020, 95% CI (0.793, 1.248), *SE* = 0.116, *p* < 0.001] and correctional settings as well [*b* = 1.026, 95% CI (0.451, 1.601), *SE* = 0.293, *p* < 0.001].

Next, a random effects meta-analysis (*k* = 51) with moderators was examined. The mixed-effects model showed a significant effect on risk factors [*b* = 0.898, 95% CI (0.550, 1.245), *SE* = 0.177, *p* < 0.001] and on protective factors as well [*b* = 1.080, 95% CI (0.833, 1.328), *SE* = 0.126, *p* < 0.001]. The between-study heterogeneity was considerable [*Q*_(df = 49)_ = 254.079, *p* < 0.001, *I*^2^ = 84.20%].

Subsequently, analyses were done for each category of the risk and protective factors separately.

##### 3.5.2.1. Addiction

**Figure 6:**
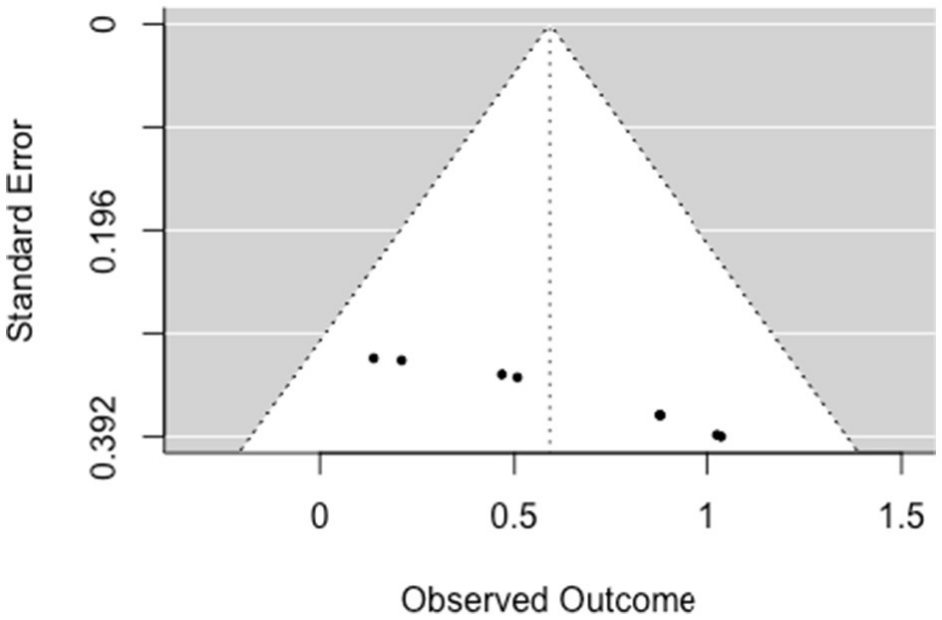
Funnel plot for addiction (pre-post studies).

The multilevel random-effects meta-analysis (*k* = 8) indicated an overall significant effect on addiction [*b* = 0.591, 95% CI (0.340, 0.842), *SE* = 0.128, *p* < 0.001]. The between-study heterogeneity was very low [*Q*_(df = 7)_ = 7.343, *p* = 0.394; *I*^2^ = 6.09%]. Egger's test gave *z* = 2.67 (*p* = 0.008), which indicated funnel plot asymmetry (Figure 6). This concerned effects on addiction related thoughts, and not directly on substance abuse since it was in a controlled environment.

##### 3.5.2.2. Psychiatric symptoms

**Figure 7:**
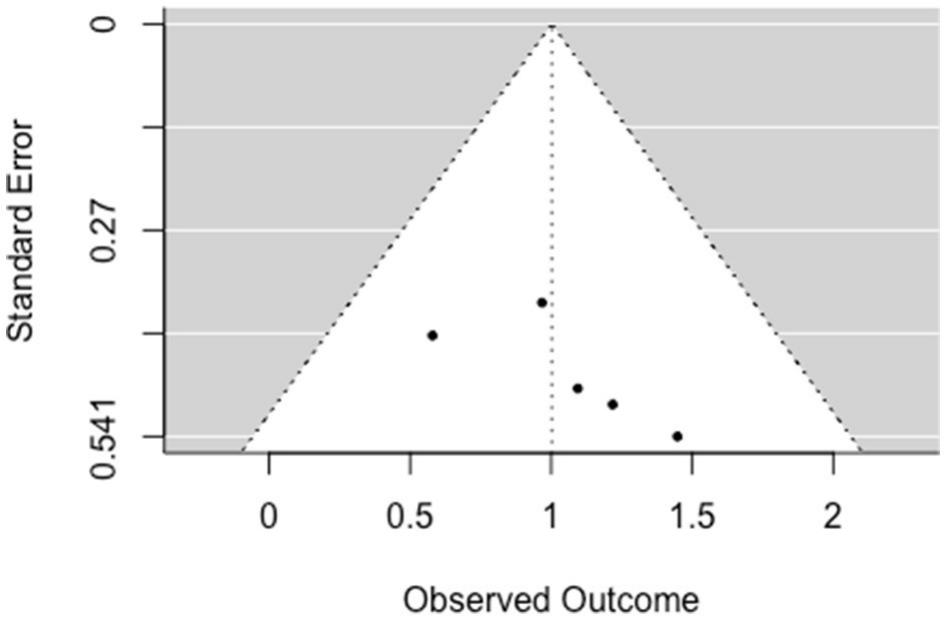
Funnel plot for psychiatric symptoms (pre-post studies).

The multilevel random-effects meta-analysis (*k* = 5) indicated an overall significant effect on psychiatric symptoms [*b* = 1.002, 95% CI (0.612, 1.392), *SE* = 0.199, *p* < 0.001]. The between-study heterogeneity was very low [*Q*_(df = 4)_ = 0.739, *p* = 0.739; *I*^2^ = 0.00%]. Egger's test gave *z* = 1 (*p* = 0.32), which indicated no funnel plot asymmetry (Figure 7).

##### 3.5.2.3. Social-functioning

**Figure 8:**
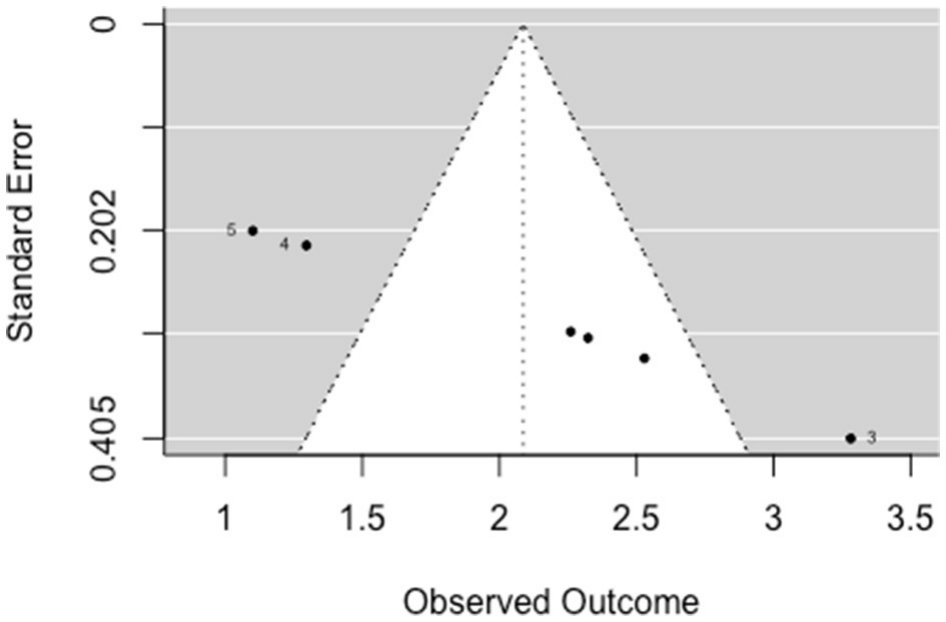
Funnel plot for social functioning (pre-post studies). Effect size outside the 95% confidence interval around 0 are labeled with their row number in the data set.

The multilevel random-effects meta-analysis (*k* = 6) indicated a significant effect on social functioning [*b* = 2.087, 95% CI (1.440, 2.733), *SE* = 0.330, *p* < 0.001]. The between-study heterogeneity was considerable [*Q*_(df = 5)_ = 40.798, *p* < 0.001; *I*^2^ = 88.07%]. Egger's test gave *z* = 6.38 (*p* = 0), which indicated funnel plot asymmetry (Figure 8).

##### 3.5.2.4. Psychological functioning

**Figure 9:**
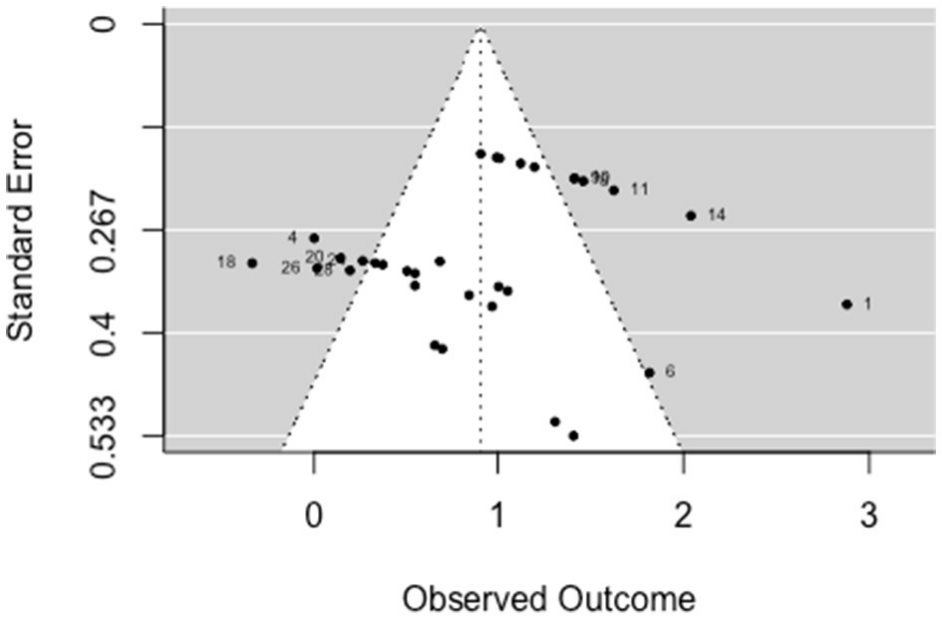
Funnel plot for psychological functioning (pre-post studies). Effect sizes outside the 95% confidence interval around 0 are labeled with their row number in the data set.

The multilevel random-effects meta-analysis (*k* = 32) indicated an overall significant effect on psychological functioning [*b* = 0.908, 95% CI (0.680, 1.135), *SE* = 0.116, *p* < 0.001]. The between-study heterogeneity was considerable [*Q*_(df = 31)_ = 151.429, *p* < 0.001; *I*^2^ = 82.71%]. Based on visual inspection there was no evidence of funnel plot asymmetry. Egger's test gave *z* = −0.41 (*p* = 0.679), which indicated no funnel plot asymmetry (Figure 9).

## 4. Discussion

The present study focused on effectiveness of the arts therapies in forensic care, included 23 studies. The results of the narrative synthesis showed that the arts therapies make use of hypothesized mechanisms of change which can be described as a number of regulatory processes that are shaped through the specific creative means of each form of arts therapy: - perceptive awareness (intero- and exteroception); - emotion regulation; - stress regulation; - impulse regulation; - cognitive regulation; - social regulation; - self-expression; - behavior regulation; and - self management. Improvement of these regulatory processes through arts therapies may lead to improvements of protective factors such as psychological and social functioning, and may possibly (more indirectly) contribute to decrease of risk factors, such as, psychiatric symptoms and addiction. These changes may contribute to lowering the risk of re-offending, which needs to be investigated in future studies.

The results of the meta-analysis based on 18 included studies showed significant effects of the arts therapies on risk factors as well as protective factors. Of the controlled studies, significant outcomes were shown on psychiatric symptoms, social functioning and psychological functioning, but not on criminal/antisocial behavior, due to insufficient data. Of the pre-post studies, significant outcomes were shown on addiction, psychiatric symptoms, social functioning and psychological functioning. In the RCT/CCT studies, art therapy and music therapy showed significant outcomes. Drama therapy and dance movement therapy both concerned only one study, so no conclusions can be drawn for these intervention types. Of pre-post designs, art therapy, music therapy and drama therapy studies were included and all intervention types indicated significant outcomes. Art therapy concerned only one study, so no conclusion can be drawn for this intervention type. All setting types showed significant improvements as well, indicating that arts therapies are effective in forensic care as well as in correctional settings.

The relationship between the hypothesized mechanisms and the effects found in the meta-analyses can be illustrated with some examples. The arts therapies use art, music, dance or drama to create experiences. Many of the interventions found in the included studies are aimed at experiencing the *self* , and then also in relation to others. The self can be seen as a central aspect of the individual that plays a role in motivation, cognition, affect and emotion and social identity (66). Through interoception—the process of sensing bodily signals—social connection is targeted (67). Improving interoceptive abilities through arts therapies may be key for improving social connection. The meta-analyses also show that arts therapies are effective in improving social functioning. Emotion regulation is targeted as well. By exploring emotions, learning to feel and learning to correctly label these feelings through experiences in the arts, interoception and introspection are practiced. Arts therapies emphasize guiding the patient's attention to interoceptive, kinesthetic, and proprioceptive experience. This kind of inner attention, in addition to imagery can lead to the resolution of symptoms of chronic and traumatic stress (68). Using these sensations might be an effective therapeutic tool. A higher interoceptive accuracy and awareness might lead to improved impulse control. Also in this aspect, the meta-analyses show that the art therapies have effect. This is particularly relevant for the forensic setting, as impulse control is an important predictor of criminal and antisocial behavior [e.g., (69)].

A strength of this study is that this is the first in this field to provide an overview of studies, a narrative synthesis of mechanisms of change as well as a meta-analysis of results of the arts therapies in forensic care. This study used a thoroughly systematic approach following strict criteria. With this study we contribute to the knowledge base of arts therapies in forensic care. We present an overarching framework of regulatory processes based on the narrative synthesis of mechanisms.

This study has also some limitations to consider. First, only a limited number of the studies included in this review were of strong methodological quality. This is often the conclusion of literature reviews concerning psychological interventions, because assessment tools have not been primarily developed for studies of these types of interventions where blinding is not possible [e.g., (70)]. A second limitation is that some of the included studies report a relatively high dropout rate. This might be caused by the nature of the setting (when duration of stay does not depend on the course of therapy, but on other matters) and characteristics of the population; it concerns involuntary detained individuals with psychiatric problems for whom motivation and adherence to treatment is not naturally. A third limitation is that the studies are very heterogeneous in type of outcome measures and intervention characteristics. Furthermore, in a few cases, there were only two studies in the analysis. These analyses should therefore be seen as exploratory, as a first step. Therefore, we first investigated the total set of outcomes and the involved moderators, followed by exploratory analyses of clusters of outcomes. Hence, we cannot make strong statements about the effects. However, results are visible on both protective and risk factors. Also, IQ might be an important factor given the characteristics of the forensic population in general. IQ measures were only present in one of the studies and therefore IQ was not part of the meta-analysis. A last limitation is that, based on the present review/meta-analysis, no conclusions on the effectiveness of arts therapies on addiction in forensic care could be drawn because of lack of studies. However, there are publications on arts therapies therapy for addiction problems [e.g., (71–74)], but these studies concern no forensic setting or are not effect studies.

A recent report (23) describes two priorities for future research for arts therapies in forensic psychiatry: formulating mechanisms of change of arts therapies and describing art therapeutic interventions in forensic care. This study contributes to these priorities and provides an impetus for further elaboration and deepening. An example of a potential working mechanism of arts therapies is the better regulation of emotions. As a result, individuals learn to better manage their anger, which reduces the risk of recidivism and leads to recovery from psychiatric problems. For a targeted and relevant use of arts therapies for the purposes of forensic treatment, it is important to properly recognize what works and how it works.

Because there are no other reviews or meta-analyses in this particular area our results are a first step in gaining insight in recent evidence-based knowledge about effects and mechanisms in forensic care. Our findings are in line with a previous practice-based study (24) which investigated arts therapies in the Dutch forensic psychiatric practice focusing specifically on aggression problems. In this study the different regulatory processes that were found in the present study can be recognized. This study mapped out which indications, objectives, interventions and (observed) effects were mentioned by the different arts therapists in forensic psychiatry. It pointed out different indications, with an overlap between the different arts therapies. For example, drama therapy is aiming at aggression regulation based on the mechanism to trigger responses, learning self-control and distancing oneself by fictional roles and scenes. Music therapy was indicated to develop recognition of emotions using the mechanism of self-expression through music. Art therapy was aimed at gaining insight in thoughts, beliefs, feelings, behavior and problems using the mechanism of imaging. Dance therapy was also aimed at anger regulation through body and movement expression, using controlled force to transform destructive into constructive behavior.

Another consideration is that about 40% of the people in the criminal justice system have a mild to borderline intellectual disability and an estimate of 20% has MID (75, 76). This is more than twice as much as in the general population. With the right treatment and guidance, the chance of recurrence of criminal behavior is reduced. Because of their experiential, less verbal approach, arts therapies may be relevant for this target group. However, IQ measures were present in only one of the studies (52). It is recommended to describe this in future studies.

Future research should examine effects of the arts therapies more sound using strong research designs, adequate sample sizes and strict quality criteria. Only with studies with a strong rigor there can be built on an evidence base for the arts therapies. Furthermore, mechanisms of change should be clearly defined as the definition varies. Also, mechanisms are often only implicitly mentioned. For building a theory base mechanisms of change should be described and studied more specifically. The articulated central regulatory processes can be a helpful guideline in developing and underpinning practice-based interventions in clinical practice and as a framework for future research. We recommend practitioners and researchers to use this overarching framework as a shared language for communication and building a shared knowledge base.

In conclusion, the analyses in this study should be considered exploratory. More research is needed to gain more solid conclusions about the effectiveness and mechanisms of arts therapies in forensic care. However, this is a first systematic review, synthesis of mechanisms and meta-analysis in this field. The results of these analyses are promising and show effects of arts therapies on risk and protective factors.

## Author contributions

AA, SH, and SvH made a substantial contribution to the design of the article, conduction of the study and the different analyses, and interpretation of data for the article. SN and AA prepared quantitative data for the meta-analysis. PV designed the model, the computational framework for the meta-analysis, and performed the analyses. AA and SH wrote the manuscript in consultation of SvH. SvH supervised the overall project. All authors contributed to the article and approved the submitted version.

## Supplementary material

Supplementary material 1 Search strings.

Supplementary material 2 Data-extraction sheet.

Supplementary material 3 Analysis script for meta-analysis RCT/CCT.

Supplementary material 4 Analysis script for meta-analysis pre-post studies.

Supplementary material 5 Excluded full texts with reasons.

Supplementary material 6 Quality assessment of included studies.

Supplementary material 7 Intervention characteristics and mechanisms of change.

Supplementary material 8 PRISMA checklist.
