# Supplementary material for: Effectiveness and mechanisms of the arts therapies in forensic care. A systematic review, narrative synthesis, and meta analysis
Source: Front Psychiatry. 2023 May 19;14:1128252. doi: 10.3389/fpsyt.2023.1128252 (PMC10235769; doi:10.3389/fpsyt.2023.1128252)
Supplement: Supplementary material 1 — Search strings. [file Data_Sheet_1.DOCX]

Search terms

*Intervention:*

“arts therap*” OR “arts psychotherap*”

“art therap*” OR “art psychotherapy**” OR “creative arts therap*”

“music therap*” OR “musictherap*”

“drama therap*” OR “dramatherap*” OR “psychodrama” OR “drama psychotherapy*” OR

“dance therap*” OR “dancetherap*” OR “dance movement therap*” OR “dance movement psychotherapy*” OR “Therap* movement” OR “Therap* dance OR Dance-therap*” OR (“Expressive movement AND therap*”) OR (“Expressive dance AND therap*”) OR “Creative movement therap*” OR “Creative dance therap*”

*Study population:*

“offender*” OR “criminal” OR “forensic” OR “prison” OR “delinquen*” OR “detention” OR “imprisoned” OR “inmate*” OR “crime” OR “convict*” OR “detainee*” OR “recidivist*” OR “justice” OR “violence” OR “abuse” OR “recidivism” OR “theft” OR “rape” OR “homicide” OR “assault OR “gangs” OR “sexual abuse” OR “pyromania” OR “predelinquent” OR “conduct disorder” OR “CD” OR “oppositional” OR “ODD” OR “disruptive behaviour” OR “aggression” OR “antisocial” OR “anger” OR “hostility” OR “outrage” OR “threat*” OR “kleptomania” OR “self-destructive” OR “harassment” OR “vandalism” OR “coercion” OR “deviant behaviour” OR “ADHD” OR “risk taking behaviour” OR “frustration (tolerance)” OR “externalising behaviour” OR “impulse control disorder*” OR “attachment disorder*” OR “poverty” OR “low socio-economic status” OR “low SES” OR “trauma” OR “substance abuse” OR “learning difficulties” OR “intellectual disability” OR “mental disability” OR “mentally challenged”
