# Supplementary material for: Effectiveness and mechanisms of the arts therapies in forensic care. A systematic review, narrative synthesis, and meta analysis
Source: Front Psychiatry. 2023 May 19;14:1128252. doi: 10.3389/fpsyt.2023.1128252 (PMC10235769; doi:10.3389/fpsyt.2023.1128252)
Supplement: Supplementary material 3 — Analysis script for meta-analysis RCT/CCT. [file Data_Sheet_3.PDF]

Results meta analysis

```
r format(Sys.time(), '%Y-%m-%d')
```

```{r echo=FALSE, warning=FALSE, message = FALSE} library(knitr) opts_chunk$set(fig.path='figure/graphics-', cache.path='cache/graphics-', fig.align='center', external=TRUE, echo=TRUE, warning=FALSE, fig.pos='H' ) a4width<- 8.3 a4height<- 11.7

library(metafor)

load("data12.rda") data12 <- data12[!is.na(data12$yi),] range12 <- range(table(data12$studyNumber))

```
Data RCT design

First all records with design = "1" (RCT) or "2" (CCT) were selected.

When the mean at T2 of the experimental group was missing, it was
imputed with the value of the mean of the experimental group at T3, if
this existed. Similarly, the SD at T1 was used to impute the missing
SD at T2 for the experimental group. The same kind of imputation was
done for the control group.

Next, based on the means and SD's of the control and experimental
group at T2, an effect size ("SMD") and variance of the effect size
were computed using the `escalc` function from the `metafor` package
(Viechtbauer, 2010) and these variables were added to the data. The
"SMD" is the standardized mean difference (Hedges, 1981).
All effects were coded such that a positive effect size implies an
effect in accordance with the expected effect of the intervention.

There were `r sum(!is.na(data12[,"yi"]))` records in this data set
with non-missing effect sizes.

Random effects models

Next, a meta analysis with random effects using the `metafor` package
was run on these data. There were `r
length(unique(data12$studyNumber))` studies included in the data, with
the number of effect sizes within each study running from `r
range12[1]` to `r range12[2]`.

The first analyses were done on the complete sample. First without
```
```

```
moderators and in addition, risk factor (protective versus risk),
intervention type and the setting (forensic-psychiatric, forensic,
other) were separately included as moderators.
```

```
```{r analysis3, eval=TRUE, echo=F, message=FALSE, warning=FALSE,
include=TRUE, comment=" "}
## Random effect model with moderators

res3all <- metafor::rma(yi , vi,
                       data=data12)

res3a <- metafor::rma(yi , vi,
                       mods = ~ - 1 + studyNumber,
                       data=data12)
res3b <- metafor::rma(yi , vi,
                       mods = ~ - 1 + outcomeType,
                       data=data12)

res3c <- metafor::rma(yi, vi,
                       mods =  ~ -1  + ivType,
                       data=data12)

res3d <- metafor::rma(yi , vi,
                       mods = ~ - 1  + setting,
                       data=data12)


summary(res3all, digits=3)
#summary(res3a, digits=3)
summary(res3b, digits=3)
summary(res3c, digits=3)
summary(res3d, digits=3)
```

Analyses per group

Next, the analyses were done for each category of the risk factor separately (without moderators).

```{r analysis2, eval=TRUE, echo=FALSE, message=FALSE, warning=FALSE, include=TRUE, comment=" "}

## Random effect model

rct1 <- subset(data12, Riskfactor == “criminal/antisocial”) rct2 <- subset(data12, Riskfactor == “psychiatric”) rct3 <- subset(data12, Riskfactor == “soc-funct”) rct4 <- subset(data12, Riskfactor == “psy-funct”)

res1 <- metafor::rma(yi, vi, data=rct1) res2 <- metafor::rma(yi, vi, data=rct2) res3 <- metafor::rma(yi, vi, data=rct3) res4 <- metafor::rma(yi, vi, data=rct4)

a1 <- summary(res1, digits=3) a2 <- summary(res2, digits=3) a3 <- summary(res3, digits=3) a4 <- summary(res4, digits=3)

egg1 <- metafor::regtest(res1) egg2 <- metafor::regtest(res2) egg3 <- metafor::regtest(res3) egg4 <- metafor::regtest(res4)

```
Criminal/antisocial

The model results show that the overall effect `r round(coef.rma(res1)
[1], 3)` is `r ifelse(res1$pval < 0.05, "", "not")` significant. The
between-study heterogeneity is `r round(res1$I2, 1)`%.

```{r results1a, eval=TRUE, echo=F, message=FALSE, warning=FALSE,
include=TRUE, comment=" "}

a1
metafor::forest.rma(res1, addpred=T, showweights = T)
```

The underlying true effects were heterogeneous. Next the funnel plot for this analysis is shown. Effect sizes outside the 95% confidence interval around 0 are labelled with their row number in the data set.

```{r results1b, eval=TRUE, echo=F, message=FALSE, warning=FALSE, include=TRUE, comment=" "}

metafor::funnel.rma(res1, label=“out”, cex=0.5)

```
Egger's test gave z = `r round(egg1$zval,2)` (p = `r
round(egg1$pval,3)`), which indicated `r ifelse(egg1$pval < 0.05, "",
"no")` funnel plot asymmetry.

Psychiatric

The model results show that the overall effect `r round(coef.rma(res2)
[1], 3)` is `r ifelse(res2$pval < 0.05, "", "not")` significant. The
between-study heterogeneity is `r round(res2$I2, 1)`%.

```{r results2a, eval=TRUE, echo=F, message=FALSE, warning=FALSE,
```
```

```
include=TRUE, comment=" "}

a2
metafor::forest.rma(res2, addpred=T, showweights = T)
```

The underlying true effects were heterogeneous. Next the funnel plot for this analysis is shown. Effect sizes outside the 95% confidence interval around 0 are labelled with their row number in the data set.

```{r results2b, eval=TRUE, echo=F, message=FALSE, warning=FALSE, include=TRUE, comment=" "}

metafor::funnel.rma(res2, label="out", cex=0.5)

```
Egger's test gave z = `r round(egg2$zval,2)` (p = `r
round(egg2$pval,3)`), which indicated `r ifelse(egg2$pval < 0.05, "",
"no")` funnel plot asymmetry.

Social-functioning

The model results show that the overall effect `r round(coef.rma(res3)
[1], 3)` is `r ifelse(res3$pval < 0.05, "", "not")` significant. The
between-study heterogeneity is `r round(res3$I2, 1)`%.

```{r results3a, eval=TRUE, echo=F, message=FALSE, warning=FALSE,
include=TRUE, comment=" "}

a3
metafor::forest.rma(res3, addpred=T, showweights = T)
```

The underlying true effects were heterogeneous. Next the funnel plot for this analysis is shown. Effect sizes outside the 95% confidence interval around 0 are labelled with their row number in the data set.

```{r results3b, eval=TRUE, echo=F, message=FALSE, warning=FALSE, include=TRUE, comment=" "}

metafor::funnel.rma(res3, label="out", cex=0.5)

```
Egger's test gave z = `r round(egg3$zval,2)` (p = `r
round(egg3$pval,3)`), which indicated `r ifelse(egg3$pval < 0.05, "",
"no")` funnel plot asymmetry.

Psychological-functioning

The model results show that the overall effect `r round(coef.rma(res4)
[1], 3)` is `r ifelse(res4$pval < 0.05, "", "not")` significant. The
```
```

```
between-study heterogeneity is `r round(res4$I2, 1)`%.

```{r results4a, eval=TRUE, echo=F, message=FALSE, warning=FALSE,
include=TRUE, comment=" "}

a4
metafor::forest.rma(res4, addpred=T, showweights = T)
```

The underlying true effects were not heterogeneous. Next the funnel plot for this analysis is shown. Effect sizes outside the 95% confidence interval around 0 are labelled with their row number in the data set.

```{r results4b, eval=TRUE, echo=F, message=FALSE, warning=FALSE, include=TRUE, comment=" "}

metafor::funnel.rma(res4, label="out", cex=0.5)

```

Egger's test gave z = `r round(egg4$zval,2)` (p = `r round(egg4$pval,3)`), which indicated `r ifelse(egg4$pval < 0.05, "", "no")` funnel plot asymmetry.
```
