# Supplementary material for: Effectiveness and mechanisms of the arts therapies in forensic care. A systematic review, narrative synthesis, and meta analysis
Source: Front Psychiatry. 2023 May 19;14:1128252. doi: 10.3389/fpsyt.2023.1128252 (PMC10235769; doi:10.3389/fpsyt.2023.1128252)
Supplement: Supplementary material 4 — Analysis script for meta-analysis pre-post studies. [file Data_Sheet_4.PDF]

Results meta analysis of pre-post designs

`r format(Sys.time(), '%Y-%m-%d')`

```{r echo=FALSE, warning=FALSE, message = FALSE} library(knitr) opts_chunk$set(fig.path='figure/graphics-', cache.path='cache/graphics-', fig.align='center', external=TRUE, echo=TRUE, warning=FALSE, fig.pos='H' ) a4width<- 8.3 a4height<- 11.7

library(metafor)

load("data3.rda")

## remove studies after consultation df <- data3[!is.na(data3[,"yi"]),] df <- df[!(df $mean_T 1_e x p_g r o u p=$¿7.67 $\vee df$ mean_T1_exp_group== 10.22),] s <- df$studyNumber==12 & df$mean_T1_exp_group %in% c(5.19,5.26,4.82,5.09, 5.24) df <- df[!s,]

range3 <- range(table(df$studyNumber))

```
Data Pre-post design

First all records with design = "3" (pre-post design) were selected.
When the mean at T2 of the experimental group was missing, it was
imputed with the value of the mean of the experimental group at T3, if
this existed. Similarly, the SD at T3 was used to impute the missing
SD at T2 for the experimental group.
The correlation (r) between the two measurements was never reported.
The value of $r = 0.5$ was therefore used as a reasonable
approximation. Furthermore, when the SD's at T1 and T2 were not
reported, but the standard deviation of the change scores was directly
reported, then the SD at T1 was imputed with the SD of the change
score and the SD at T2 and the correlation were set to 0.
Next, based on the means and SD's of the experimental group at T1 and
T2 and the correlation between them, an effect size ("SMCC") and
variance of the effect size were computed using the `escalc` function
from the `metafor` package (Viechtbauer, 2010) and these variables
were added to the data. The "SMCC" is the standardized mean change
using change score standardization (Gibbons et al., 1993).
One extreme effect size (-7.4), which was due a very low SD of the
change scores, was removed. One record with a very likely incorrect
value (outlier) in the mean at T2 (value 0.14, record number 230) was
also removed.
All effects were coded such that a positive effect size implies an
effect in accordance with the expected effect of the intervention.
After close inspection 7 studies were removed, because ....

There were `r sum(!is.na(df[,"yi"]))` records in this data set with
```
```

non-missing effect sizes.

Random effect model

Next, a meta analysis with random effects using the `metafor` package was run on these data. The clustering within studies was accounted for by taking the study number as a moderator. There were `r length(unique(df$studyNumber))` studies included in the data, with the number of effect sizes within each study running from `r range3[1]` to `r range3[2]`.

The first analyses were done on the complete sample. First without moderators and in addition, risk factor (protective versus risk), intervention type and the setting (forensic-psychiatric, forensic, other) were separately included as moderators.

````
```{r analysis3, eval=TRUE, echo=F, message=FALSE, warning=FALSE,
include=TRUE, comment=" "}

## Random effect model with moderators

res3all <- metafor::rma(yi , vi,
                       data=df)

res3a <- metafor::rma(yi , vi,
                       mods = ~ - 1 + studyNumber,
                       data=df)
res3b <- metafor::rma(yi , vi,
                       mods = ~ - 1 + outcomeType,
                       data=df)

res3c <- metafor::rma(yi, vi,
                       mods =  ~ -1  + ivType,
                       data=df)

res3d <- metafor::rma(yi , vi,
                       mods = ~ - 1  + setting,
                       data=df)


summary(res3all, digits=3)
#summary(res3a, digits=3)
summary(res3b, digits=3)
summary(res3c, digits=3)
summary(res3d, digits=3)
````
````

Analyses per group

Next, the analyses were done for each category of the risk factor separately (without moderators).

```{r analysis2, eval=TRUE, echo=FALSE, message=FALSE, warning=FALSE, include=TRUE, comment=" "}

## Random effect model

table(df$Riskfactor) prepost1 <- subset(df, Riskfactor == “addiction”) prepost2 <- subset(df, Riskfactor == “psychiatric”) prepost3 <- subset(df, Riskfactor == “soc-funct”) prepost4 <- subset(df, Riskfactor == “psy-funct”)

res1 <- metafor::rma(yi, vi, data=prepost1) res2 <- metafor::rma(yi, vi, data=prepost2) res3 <- metafor::rma(yi, vi, data=prepost3) res4 <- metafor::rma(yi, vi, data=prepost4)

a1 <- summary(res1, digits=3) a2 <- summary(res2, digits=3) a3 <- summary(res3, digits=3) a4 <- summary(res4, digits=3)

egg1 <- metafor::regtest(res1) egg2 <- metafor::regtest(res2) egg3 <- metafor::regtest(res3) egg4 <- metafor::regtest(res4)

````
Addiction

The model results show that the overall effect `r round(coef.rma(res1)
[1], 3)` is `r ifelse(res1$pval < 0.05, "", "not")` significant.

```{r results1a, eval=TRUE, echo=F, message=FALSE, warning=FALSE,
include=TRUE, comment=" "}

a1
metafor::forest.rma(res1, addpred=T, showweights = T)
````

The underlying true effects were heterogeneous. Next the funnel plot for this analysis is shown. Effect sizes outside the 95% confidence interval around 0 are labelled with their row number in the data set.

```{r results1b, eval=TRUE, echo=F, message=FALSE, warning=FALSE, include=TRUE, comment=" "}

metafor::funnel.rma(res1, label=“out”, cex=0.5)

```
Egger's test gave z = `r round(egg1$zval,2)` (p = `r
round(egg1$pval,3)`), which indicated `r ifelse(egg1$pval < 0.05, "",
"no")` funnel plot asymmetry.
```
```

Psychiatric

The model results show that the overall effect `r round(coef.rma(res2)[1], 3)` is `r ifelse(res2$pval < 0.05, "", "not")` significant.

```{r results2a, eval=TRUE, echo=F, message=FALSE, warning=FALSE, include=TRUE, comment=" "}

a2
metafor::forest.rma(res2, addpred=T, showweights = T)

The underlying true effects were heterogeneous. Next the funnel plot for this analysis is shown. Effect sizes outside the 95% confidence interval around 0 are labelled with their row number in the data set.

```{r results2b, eval=TRUE, echo=F, message=FALSE, warning=FALSE, include=TRUE, comment=" "}

metafor::funnel.rma(res2, label=“out”, cex=0.5)

Egger's test gave z = `r round(egg2$zval,2)` (p = `r round(egg2$pval,3)`), which indicated `r ifelse(egg2$pval < 0.05, "", "no")` funnel plot asymmetry.

### Social-functioning

The model results show that the overall effect `r round(coef.rma(res3)[1], 3)` is `r ifelse(res3$pval < 0.05, "", "not")` significant.

```{r results3a, eval=TRUE, echo=F, message=FALSE, warning=FALSE, include=TRUE, comment=" "}

a3
metafor::forest.rma(res3, addpred=T, showweights = T)

The underlying true effects were heterogeneous. Next the funnel plot for this analysis is shown. Effect sizes outside the 95% confidence interval around 0 are labelled with their row number in the data set.

```{r results3b, eval=TRUE, echo=F, message=FALSE, warning=FALSE, include=TRUE, comment=" "}

metafor::funnel.rma(res3, label=“out”, cex=0.5)

Egger's test gave z = `r round(egg3$zval,2)` (p = `r

round(egg3$pval,3)`), which indicated `r ifelse(egg3$pval < 0.05, "", "no")` funnel plot asymmetry.

### Psychological-functioning

The model results show that the overall effect `r round(coef.rma(res4)[1], 3)` is `r ifelse(res4$pval < 0.05, "", "not")` significant.

```{r results4a, eval=TRUE, echo=F, message=FALSE, warning=FALSE, include=TRUE, comment=" "}

a4
metafor::forest.rma(res4, addpred=T, showweights = T)

The underlying true effects were heterogeneous. Next the funnel plot for this analysis is shown. Effect sizes outside the 95% confidence interval around 0 are labelled with their row number in the data set.

```{r results4b, eval=TRUE, echo=F, message=FALSE, warning=FALSE, include=TRUE, comment=" "}

metafor::funnel.rma(res4, label="out", cex=0.5)

```

Egger's test gave z = r round(egg4$zval,2) (p = r round(egg4$pval,3)), which indicated r ifelse(egg4$pval < 0.05, "", "no") funnel plot asymmetry.
```
