# Supplementary material for: Effectiveness and mechanisms of the arts therapies in forensic care. A systematic review, narrative synthesis, and meta analysis
Source: Front Psychiatry. 2023 May 19;14:1128252. doi: 10.3389/fpsyt.2023.1128252 (PMC10235769; doi:10.3389/fpsyt.2023.1128252)
Supplement: Supplementary material 5 — Excluded full texts with reasons. [file Data_Sheet_5.docx]

**Supplementary Material 5** **Excluded full texts and outcomes, with reasons**

***Reasons for exclusion of full-texts for systematic review***

Three articles had the wrong language (Kvasnicka, 1983; Legendre, 1990; Schiltz, Ciccarello, Ricci-Boyer, & Schiltz, 2013), one was published before 1980 (Haskell, 1957), nine articles had the wrong publication type (Erikson, 2009; McConnel, 1989; Schacht-Lavine, 1982; Segall, 2017; Silver, 2005; Truppi, 2005; Weide, 1986; Wylie, Shuker, & Sullivan, 2010; McClelland, 2010), one article had the wrong population/setting (Appleton, Barkley, & Katz, 1986), twelve articles were aimed at youth (Abrahams & van Dooren, 2018; Adams & Vetter, 1981; Becker, Kaplan, & Kavoussi, 1988; Gaffney & McFall, 1981; George & Kasinathan, 2015; Harber, 2011; Hartz & Thick, 2005; Lopez & Carolan, 2001; Persons, 2009; Rapp-Paglicci, Stewart, Rowe, 2009; Shelton, 2008; Tepper-Lewis, 2019).

Seven studies did not meet the criteria for arts therapies interventions (Bensimon, Einat, Gilboa, 2015; Di Lorito, Vollm, Dening, 2019; Dolling & Day, 2013; Messina, Grella, Cartier, Torres, 2010; Niven, Totterdell, Holman, Headley, 2012; Rasmussen, Donoghue, Sheehan, 2018; Taylor & Holston, 2016), ten studies had the wrong study design (Allermann, 1989; Armstrong, & Ricard, 2016; Breiner, Tuomisto, Bouyea, Gussak, Aufderheide, 2012; Davis, & Boster, 1988, 1992; Frogget, 2007; Hanes, 2008, 2005; Petermann, & Vonnahme, 1987; Schramski, & Harvey, 1983) and three studies did not have quantitative outcomes (de Guzman, et al, 2010; Ferszt et al., 2004; Tuastad, & Stige, 2015).

***Full-texts excluded for meta analysis***

Gussak 2007: same data as Gussak 2006

Gussak 2009b: includes only between-gender data from same sample as Gussak, 2009a (Men vs Women)

Gold 2020: no effect study with pre- and post treatment measures: measures of criminal ‘events’ after six year FU; recidivism data, no effect size possible

Keulen- de Vos 2017: evoking emotional states; no effect study with pre- and post treatment measures; study type not appropriate for meta analysis

Van den Broek 2011: appearance of modi during sessions, no effect study with pre- and post treatment measures

***Outcomes excluded for meta analysis:***

Kellet 2019: FU outcomes excluded; Circle outcomes excluded (judgement by staff)

Gussak 2004/ 2006 / 2009a: FEATS excluded (non-validated measure)

Thaut: totals per outcome and the three groups together were excluded
